# Induced pluripotent stem cell modelling of HLHS underlines the contribution of dysfunctional NOTCH signalling to impaired cardiogenesis

**DOI:** 10.1093/hmg/ddx140

**Published:** 2017-05-17

**Authors:** Chunbo Yang, Yaobo Xu, Min Yu, David Lee, Sameer Alharti, Nicola Hellen, Noor Ahmad Shaik, Babajan Banaganapalli, Hussein Sheikh Ali Mohamoud, Ramu Elango, Stefan Przyborski, Gennadiy Tenin, Simon Williams, John O’Sullivan, Osman O Al-Radi, Jameel Atta, Sian E. Harding, Bernard Keavney, Majlinda Lako, Lyle Armstrong

**Affiliations:** 1 Institute of Genetic Medicine, Newcastle University, Newcastle, UK; 2 Princess Al Jawhara Al-Brahim Center of Excellence in Research of Hereditary Disorders, King Abdulaziz University, Saudi Arabia; 3 NHLI, Faculty of Medicine, Imperial College London, London, UK; 4 Department of Bioscience, Durham University, Durham, UK; 5 Division of Cardiovascular Sciences, School of Medical Sciences, Faculty of Biology, Medicine and Health, University of Manchester, Manchester, UK; 6 The Newcastle upon Tyne NHS Hospital, UK; 7 Department of Surgery, King Abdulaziz University, Saudi Arabia; 8 Central Manchester University Hospitals NHS Foundation Trust, Manchester Academic Health Science Centre, Manchester, UK

## Abstract

Hypoplastic left heart syndrome (HLHS) is among the most severe forms of congenital heart disease. Although the consensus view is that reduced flow through the left heart during development is a key factor in the development of the condition, the molecular mechanisms leading to hypoplasia of left heart structures are unknown. We have generated induced pluripotent stem cells (iPSC) from five HLHS patients and two unaffected controls, differentiated these to cardiomyocytes and identified reproducible *in vitro* cellular and functional correlates of the HLHS phenotype. Our data indicate that HLHS-iPSC have a reduced ability to give rise to mesodermal, cardiac progenitors and mature cardiomyocytes and an enhanced ability to differentiate to smooth muscle cells. HLHS-iPSC-derived cardiomyocytes are characterised by a lower beating rate, disorganised sarcomeres and sarcoplasmic reticulum and a blunted response to isoprenaline. Whole exome sequencing of HLHS fibroblasts identified deleterious variants in *NOTCH* receptors and other genes involved in the NOTCH signalling pathway. Our data indicate that the expression of *NOTCH* receptors was significantly downregulated in HLHS-iPSC-derived cardiomyocytes alongside NOTCH target genes confirming downregulation of NOTCH signalling activity. Activation of NOTCH signalling via addition of Jagged peptide ligand during the differentiation of HLHS-iPSC restored their cardiomyocyte differentiation capacity and beating rate and suppressed the smooth muscle cell formation. Together, our data provide firm evidence for involvement of NOTCH signalling in HLHS pathogenesis, reveal novel genetic insights important for HLHS pathology and shed new insights into the role of this pathway during human cardiac development.

## Introduction

Hypoplastic left heart syndrome (HLHS) is a severe combination of congenital cardiac malformations characterised by under development of the left-sided cardiac structures, including hypoplasia of the left ventricle, stenosis or atresia of the aortic and mitral valves and hypoplasia of the ascending aorta and aortic arch, resulting in a circulatory system reliant on the right ventricle only (1). HLHS occurs in 0.03% of neonates and is fatal without immediate post-partum palliative surgery (2). Prognosis is generally poor with many patients requiring a heart transplant due to congestive cardiac failure. The use of allogeneic stem cell transplantation as a therapy for HLHS has been studied in several clinical trials, but found to produce only modest improvements in left ventricular function (3–5). A more detailed understanding of the pathogenesis of HLHS is needed in order for effective treatments to be developed. A greater understanding of genetic and/or epigenetic contributions to the disease during embryonic development is central to this need.

There is a substantial familial predisposition to HLHS (6) but the genetic determinants of the disease are largely unknown. The aetiology of HLHS is thought to be multifactorial, attributed to a combination of complex inheritance and environmental influences (7). There is evidence from the literature that HLHS may result from embryonic abnormalities of blood flow (8,9) especially since the most severe presentations of the disease occur in those patients with mitral and aortic atresia. However since not all neonates born with HLHS suffer from valvular or outflow tract malformation (8), it has been hypothesised that dysfunction of genetic networks specific to the left ventricular chamber and outflow tract are also contributory to the pathogenesis of the condition. In support of this notion, it has been shown that loss-of-function mutations in the gene *HAND1* (which is expressed in left-sided cardiac structures including the left ventricular myocardium) is associated with HLHS (10). *HAND1* participates in a core transcriptional regulatory network coupled to *NKX2.5* and *NOTCH1* (11–13). *NOTCH1* has been linked to HLHS owing to its contribution to a Mendelian form of calcific aortic valve disease (12,14–16) and the identification of pathogenic compound heterozygous *NOTCH1* mutations in HLHS patients (15,17). A variety of other gene mutations or differential expressions have been identified to be associated with HLHS, including cardiac transcription factors *NKX2.5* (18), *TBX5* (19), *ETS1* (20), *FOXC1/FOXC2* (21,22), cell adhesion molecule *PECAM-1* (23) and cardiac gap junction protein *GJA1* (24). However, any myocardial susceptibility component is as yet undefined. Chromosomal disorders are also reported to be associated with HLHS. 10% of all infants born with a terminal 11q deletion (Jacobsen syndrome) have HLHS (25). Hinton *et al.* reported that HLHS links to chromosome regions 10q22 and 6q23 and is genetically related to bicuspid aortic valve (BAV) (26). The observation that bicuspid aortic valve (BAV) is more common in pedigrees containing a HLHS proband than in the general population has led to the suggestion that HLHS is a severe form of valvular malformation; however the extreme discrepancy between the incidence rates of BAV and HLHS argues against the idea that BAV is a major cause of HLHS. Although families with a case of HLHS frequently also have cases of BAV, very few families containing a case of BAV also contain a case of HLHS. This could be explained by the co-occurrence of a genetic predisposition to a mild and/or embryologically transient left-sided obstructive lesion (which could be caused by relatively common alleles, given the prevalence of bicuspid aortic valve in the population) and a rare - and possibly *de novo -* genetic predisposition to reduced myocardial growth in the presence of mildly or transiently reduced flow. This could produce the HLHS phenotype in family members with both genetic predisposing factors, whilst other family members without the ‘second hit’ impacting myocardial development would manifest only the residuum of the transient obstruction (BAV).

The rarity of HLHS renders large-scale genetic epidemiological studies unfeasible (most investigations in the literature involve less than 40 HLHS probands). Gene expression studies of human samples attempting to identify causative pathways have been restricted to the study of the right ventricle (since the left ventricle does not develop in HLHS) in postnatal life (27). It is therefore unclear whether the expression differences that have been described between HLHS and normal hearts are relevant to left ventricle chamber under development, or merely the consequence of right ventricle overload during development. In this context, we believe the use of induced pluripotent stem cell (iPSC) technology is a particularly appropriate approach to disease modelling which enables the study of earliest stages of embryonic cardiac development in patient specific cardiomyocytes. Indeed, our group and few others have performed modelling studies in iPSC-derived cardiomyocytes from HLHS patients, and have shown that HLHS iPSC have a reduced ability to differentiate into cardiomyocytes (12,17,28) and that these show a multitude of molecular and functional deficiencies when compared to equivalent cells derived from unaffected controls. The study of Gaber *et al.* (29) is interesting in that it implicates both TGFβ signalling and premature senescence of cardiomyocyte progenitor cells. Another study implicates transcriptional repression of *NKX2-5, HAND1*, and *NOTCH1* (12), thus it is plausible that multiple gene or signalling pathway dysfunctions may contribute to HLHS.

To investigate whether common genetic dysfunctions contribute to HLHS, we isolated iPSC from five HLHS patients and age matched unaffected controls and differentiated these to cardiomyocytes. Using a combination of cellular and molecular assays and next generation sequencing, we were able to identify reproducible *in vitro* cellular and functional correlates of the HLHS phenotype and we identified deleterious variants in the NOTCH signalling pathway in all HLHS patients. Reactivation of the NOTCH signalling pathway resulted in reversal of cellular phenotypes and generation of cardiomyocytes that were indistinguishable of those obtained from unaffected individuals, placing this pathway at the heart of HLHS aetiology.

## Results

### Derivation and characterisation of control and HLHS-patient specific iPSC lines

**Figure 1:**
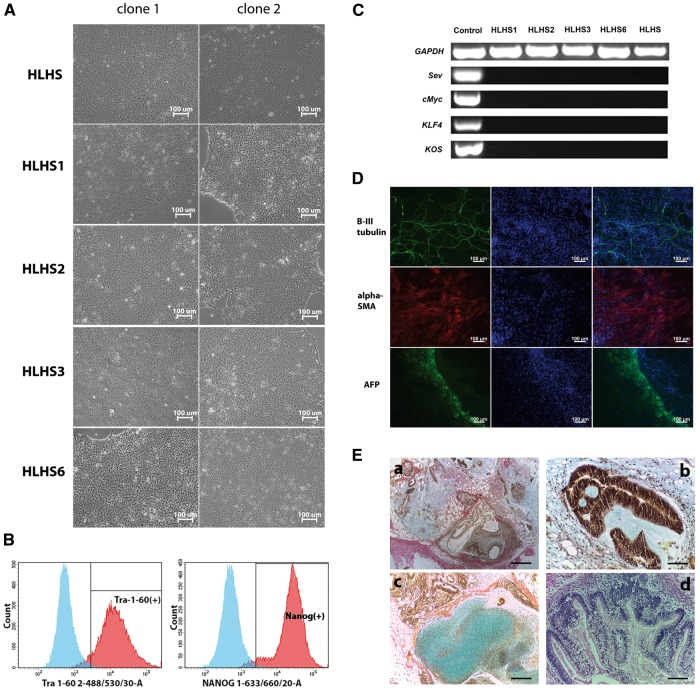
Characterization of HLHS patient specific iPSC lines. (**A**) Brightfield images of two clones derived from each patient using the non-integrative RNA based Sendai virus; (**B**) Representative example of flow cytometric analysis of key pluripotent cell markers, TRA-1-60 and NANOG. A representative example is shown from HLHS1 clone 1, however similar results were obtained for all HLHS and unaffected iPSC lines; (**C**) Elimination of Sendai viral vectors from derived iPSC lines (*KOS* stands for *KLF4*, *OCT4* and *SOX2*); (**D**) Immunofluorescent images showing antibody staining of cells derived from all three germ layers: neuronal class β-III-tubulin staining neuronal cells derived from ectoderm; α-Smooth muscle actin staining smooth muscle cells derived from mesoderm αFP (alpha-fetoprotein) staining endodermal cells. A representative example is shown from HLHS2 clone 1; however similar results were obtained for all HLHS and unaffected iPSC lines. (**E**) *In vivo* differentiation of HLHS1-clone 1 as a representative example of teratoma forming ability of HLHS patient specific iPSC lines. (A) low power showing heterogeneous structure of the teratoma; (B) neuroepithelium; (C) cartilage; (D) intestinal epithelium. (A: scale bar = 400 µm, B-D: scale bar = 50 µm).

We reported the development of an *in vitro* model of HLHS based upon the differentiation of one HLHS patient specific iPSC (named HLHS) to cardiomyocytes in a previous publication (28). In the current study, we extended the validity of this approach by deriving iPSC lines from a further four neonatal HLHS patients recruited at the Newcastle Freeman Hospital (HLHS1, 2, 3 and 6) and two unaffected controls whose dermal skin fibroblast samples were obtained from Lonza (Table 1). All iPSC clones were derived from reprogramming of dermal fibroblasts using the RNA based non-integrative Sendai virus (Thermo Fisher Cytotune 2 Kit). Since the initial control and HLHS-iPSC line was generated using a lentiviral based approach, we derived new iPSC clones from this patient fibroblasts using the Sendai virus approach. The HLHS patient specific iPSC and control iPSC lines derived from unaffected individuals showed all the hallmarks of pluripotent phenotype (Fig. 1A–C), were genetically identical to parent fibroblasts (Supplementary Material, Fig. S1B) and capable of multilineage differentiation both *in vitro* and *in vivo* (Fig. 1D and E). Several regions of loss of heterozygosity in chromosomes 3, 16 and 19 were observed in some HLHS patient fibroblasts and resulting iPSC clones (Supplementary Material, Fig. S1A) and given that they are shared between several unrelated HLHS patients, they may bear some relevance for the causes of HLHS, however this remains to be investigated further.

**Table 1:** Summary of the HLHS patients and iPSC lines derived therefrom and the unaffected controls

Name	Cell Source	Clinical Manifestation	Sex
**HLHS**	Neonatal fibroblasts- Coriell Institute for Medical Research: GM12601	Aortic and mitral atresia. Associated malformations included mild facial dysmorphism, bilateral hip dislocation, hypoplastic fingernails and mild microcephaly	Male
**HLHSI**	Neonatal fibroblasts	Mitral and aortic atresia, intact inner atrial septum	Male
**HLHS2**	Neonatal fibroblasts	Mitral and aortic atresia	Female
**HLHS3**	Neonatal fibroblasts	Mitral and aortic atresia	Male
**HLHS6**	Neonatal fibroblasts	Mitral and aortic atresia	Female
**Control SB -Neol**	Neonatal fibroblasts (Lonza)	Normal individual	Male
**Control SB-Ne03**	Neonatal fibroblasts (Lonza)	Normal individual	Male

### Differentiation of HLHS patient-specific iPSC reveals cardiomyocyte cellular, structural and functional abnormalities

**Figure 2:**
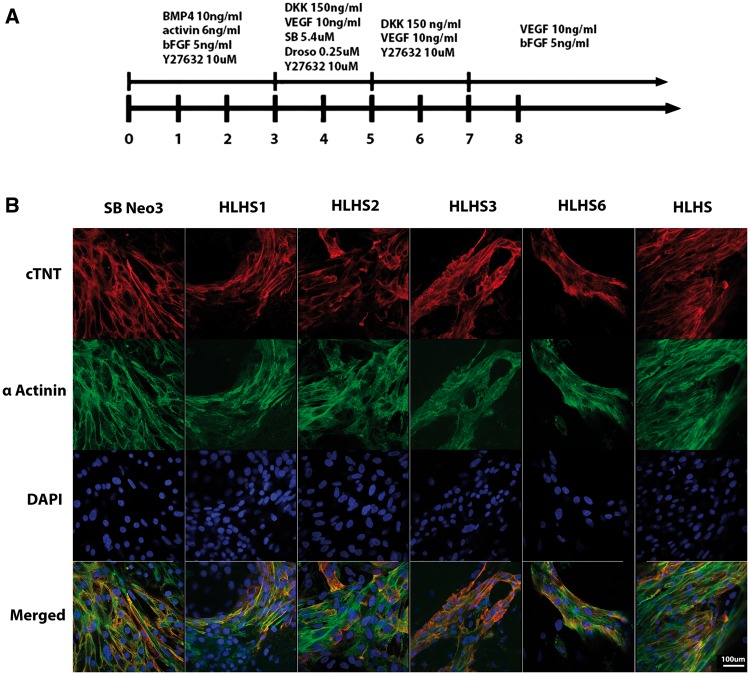
Differentiation of iPSC lines to cardiomyocytes. (**A**) Overview of the protocol used for differentiation of control and HLHS patient specific iPSC to cardiomyocytes; (**B**) Representative immunocytochemical staining of cardiomyocytes derived from control and HLHS patient specific iPSC lines using antibodies raised against alpha actinin and cardiac troponin. One clone from each patient and unaffected control is shown; however similar data were obtained for the second clone. Scale bar = 100 µm.

**Figure 3:**
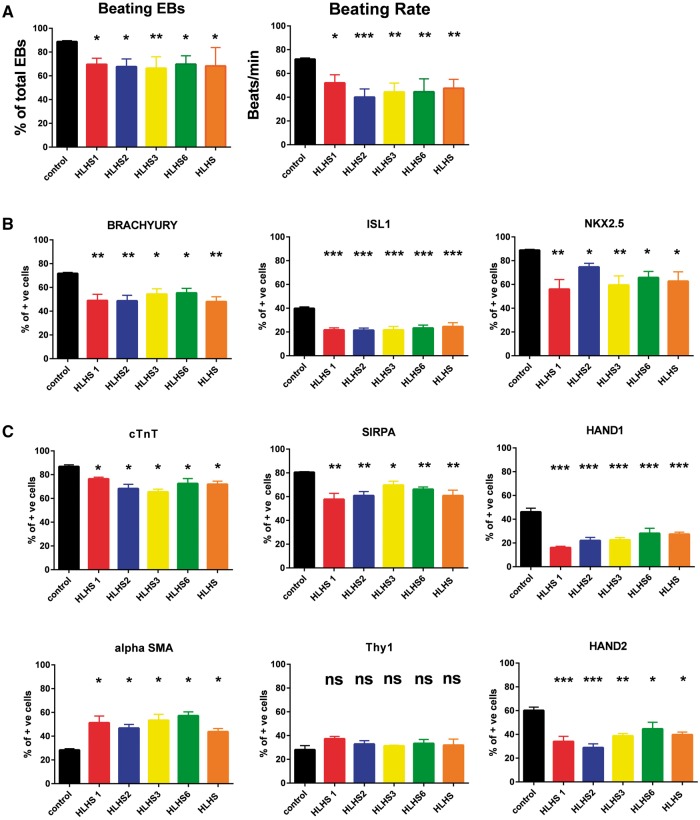
HLHS iPSC lines show an impaired ability to give rise to cardiomyocytes. (**A**) Analysis of the percentage and beating frequency of EBs that generate contraction during differentiation of control and HLHS patient specific iPSC. At least 100 EBs were assessed for each biological replicate; (**B**) Flow cytometry data analysis demonstrating a lower ability of HLHS-iPSC lines to give rise to mesodermal and cardiac progenitors when compared to control derived cells at day 7 of the differentiation time-course; (**C**) Flow cytometry data analysis demonstrating that HLHS-iPSC lines have a lower ability to give rise to cardiomyocytes but have an enhanced ability to differentiate to smooth muscle cells when compared to control derived cells at day 14 of the differentiation time-course. One way Anova analysis with Dunnett multiple comparison tests was performed. ***P < 0.001; **P value between 0.001 and 0.01; *P value between 0.01 and 0.05.HLHS iPSC lines: *N =* 6 (2 clones × triplicate biological repeats); control iPSC lines: *N =* 12 (2 clones × triplicate biological repeats × 2 unaffected control iPSC lines).

HLHS patient-specific and control iPSC lines were differentiated into cardiomyocytes (Fig. 2A and B) using the embryoid body (EB) based protocol published by Keller’s group which enables stepwise specification of mesoderm, cardiac progenitors and cardiomyocytes (30). For all patients and controls, two iPSC clones (to account for clonal variability) were differentiated into cardiomyocytes; hence all data presented in this manuscript are the average obtained from experiments conducted as biological triplicates from both clones of each HLHS patient (*n =* 6) and two unaffected controls (*n =* 12). The disease-specific iPSC lines exhibited a reduced capacity to generate spontaneously contracting cardiomyocyte clusters (Fig. 3A) and within these, the beating rate was significantly reduced (Fig. 3A), corroborating our previous findings (28). These observations may imply functional defects in cardiomyocytes derived from HLHS-iPSC.

Our flow cytometric analysis also indicated a significant difference in the ability of HLHS-iPSC to differentiate towards cells of a cardiac lineage. We observed a significant reduction in the percentage of cells expressing the mesodermal marker (BRACHYURY) and cardiomyocyte progenitor markers (ISL1, NKX2.5) at day 7 of the differentiation time course (Fig. 3B). Equally, a reduced percentage of cells expressed HAND1 and HAND2 by day 14 (Fig. 3C) as well as cardiomyocyte specific markers cTnT and SIRPA (31) was noted at both days 14 (Fig. 3C) and 21 of differentiation (Supplementary Material, Fig. S2). This reduced percentage of cells expressing cardiac progenitor and cardiomyocyte markers was observed at later differentiation time points (up to day 50), indicating that this phenomenon is not due to a delayed differentiation and suggests an impaired ability of HLHS-iPSC to undergo cardiac differentiation. As reported by Kattman *et al.* (30), no endothelial cells (identified by CD31 expression) were generated in either control or HLHS cultures following the EB differentiation protocol, suggesting that the generation of this cell type is not supported by the current culture conditions. In contrast, the percentage of cells expressing the smooth muscle marker (α-SMA) was significantly increased at both days 14 and 21 despite no change in expression of the fibroblast marker (Thy1) which typically marks the cardiac fibroblasts, suggesting a preference for smooth muscle differentiation over cardiomyocytes during HLHS-iPSC differentiation (Fig. 3C). These data were further corroborated by increased expression of the smooth muscle marker genes, *CALDESMON*, *CALPONIN* and *SMA22A* (Supplementary Material, Fig. S3). A recent report by Gaber et *al*. has highlighted activation of TGFβ signalling in HLHS foetal hearts, leading to epithelial mesenchymal transformation of cardiac fibroblasts to myofibroblasts that are characterized by some features of smooth muscle differentiation and typically express α-SMA. Gaber *et al.* went to suggest that these proliferating myofibroblast contribute to cardiac fibrosis which leads to stiffening of the ventricular walls, diminished contractility, and abnormalities in cardiac conductance (32). Despite observing increased expression of *TGF*β*1* in HLHS derived cardiomyocytes, we were unable to find increased expression of TGFβ target genes (*GDF3, NODAL;* data not shown); therefore we are inclined to speculate that increased expression of smooth muscle markers during differentiation of HLHS-iPSC to cardiac lineages most likely reflects an altered differentiation fate during iPSC differentiation.

**Figure 4:**
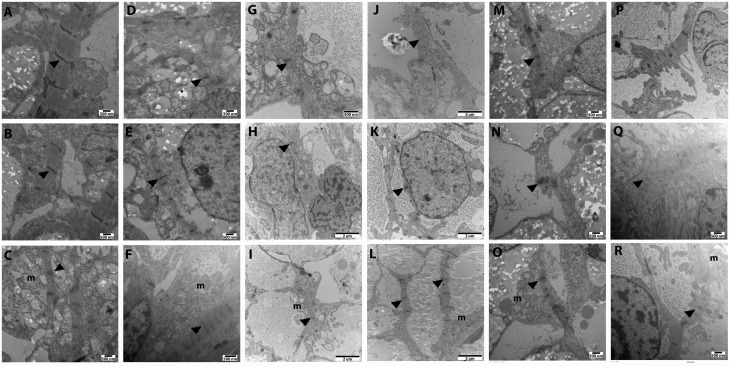
(**A–C**) Transmission electron microscopy ultrastructure of cardiomyocytes derived from an age matched control iPSC line (SB-Neo3) shows abundant myofibrillar bundles, regular transverse Z bands with intervals of around 2 µm (black arrows) and large amount of mitochondria (labeled ‘m’) similar to those observed in fetal or neonatal cardiomyocytes. (**D-R**) A more random arrangement of myofibrils with poorly defined Z bands (black arrows) was observed in HLHS-iPSC-derived cardiomyocytes. The interval between individual Z bands is frequently irregular and smaller (0.5–1.0 µm). Compared with control iPSC-CMs, HLHS-iPSC derivatives had less mitochondria (labeled ‘m’) of smaller size and mal-formed inner membrane. One clone from each HLHS patient is shown; however similar data were obtained from the second clone.

We analysed ultrastructural and functional characteristics of HLHS-specific cardiomyocytes in greater detail using transmission electron microscopy (TEM). Control iPSC- derived cardiomyocytes show well organised myofibrillar bundles with transverse Z bands (Fig. 4A–C), whereas those apparent in HLHS-iPSC-derived cardiomyocytes are less organised with poorly defined Z bands present at irregular spacing (Fig. 4D–R). In our previous publication, we studied the response of HLHS-iPSC-derived cardiomyocytes to the β1/β2 adrenergic receptor agonist isoprenaline, which normally increases the contraction frequency by enhancing Ca^2+^ ATPase activity in the sarcoplasmic reticulum (28). In this work we studied the same parameter in iPSC-CMs from two additional HLHS and one unaffected control iPSC line. Given the emergence of cells that express smooth muscle markers in during differentiation HLHS-iPSC but not control lines with the Kattman method (31), to obtain purified cultures of cardiomyocytes for electrophysiological characterisation, all iPSC lines were differentiated using a monolayer differentiation protocol that results in a high efficiency of cardiomyocyte generation (> 85% cTnT^+^, please refer to methods). The frequency of calcium transients increased in response to isoprenaline exposure in all cell lines; however the magnitude of this increase was significantly reduced in the HLHS lines compared to the control line (Supplementary Material, Fig. S4), corroborating our previous findings (28).

Furthermore, we performed RNA-seq analysis of cardiomyocytes generated from one of the HLHS patients and one unaffected control and following bioinformatic analysis we identified 163 significantly downregulated genes (corrected p value ≤ 0.05 and fold change ≥ 2.0) (Supplementary Material, Table S1). Amongst the downregulated genes were two cardiac sarcomeric proteins: (1) a ventricular isoform of myosin heavy chain (*MYH7B*) specifically expressed in heart and linked with hypertrophic cardiomyopathy and left ventricular non-compaction and (2) Troponin I 3 (*TNNI3*) expressed exclusively in cardiac muscle and already linked with both dilated cardiomyopathy and familial restrictive cardiomyopathy. The reduced expression of these two key genes supports the notion that cardiomyocyte differentiation is less efficient from HLHS-iPSC than controls. A large number of protocadherins and cadherins, encoding essential members of membrane-associated glycoproteins which are critical for cell to cell communication and regulators of intra-cellular calcium concentration were also included in this category (Supplementary Material, Table S1). Genes encoding two essential proteins, calsequestrin (*CASQ2*) and histidine rich calcium-binding protein (*HRC*) located in the sarcoplasmic reticulum and playing key roles in calcium homeostasis in cardiomyocytes were downregulated, suggesting an impaired sarcoplasmic reticulum function in HLHS cardiomyocytes. GO Biological and Molecular function as well as phenotypic searches performed using the Gene Analytics gene set analysis tool (https://ga.genecards.org/) indicated a range of processes that were affected including myocardial fibre morphology, sarcomere morphology, calcium ion binding, cell adhesion, regulation of cardiac muscle contraction by regulation of the release of sequestered calcium ion etc. which corroborate the full range of phenotypic abnormalities reported above. Since the RNA-seq experiment compared cardiomyocytes derived from one HLHS patient and one control, we performed additional qRT-PCR which confirmed downregulation of genes involved in cell adhesion, muscle contraction, actin binding proteins and myocardium fibre morphology (Supplementary Material, Fig. S5) in all HLHS samples compared to unaffected controls. Together, our data replicate the initial findings published by our group (28) and show that the multiple molecular, structural and functional differences are reproducible *in vitro* characteristics of iPSC lines derived from patients with HLHS compared to controls which implies that patient cardiomyocyte-specific factors make a substantial contribution to the development of HLHS.

### Dysfunction of the NOTCH signalling pathway underlies HLHS aetiology

**Figure 5:**
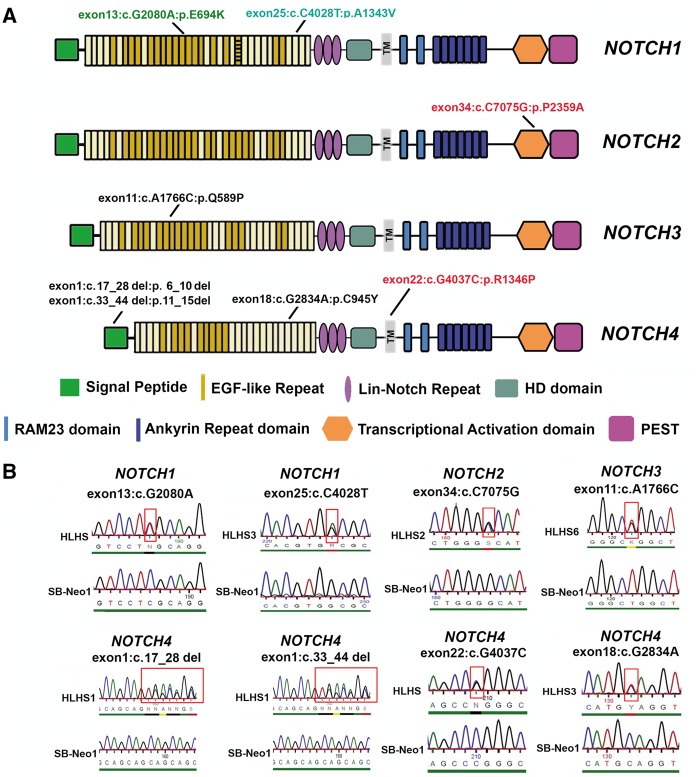
All HLHS patients harbour deleterious variants in genes involved in the *NOTCH* signalling pathway. (**A**) Summary of *NOTCH* receptor variants identified in HLHS patients. Published *NOTCH* variants associated with HLHS are shown in cyan, published variants associated with congenital heart disease are shown in green and published variants not associated with heart disease are shown in red. Novel variants uncovered by our exome analysis and not reported to date are shown in black font. (**B**) Confirmation of variants by direct sequencing in HLHS patient and unaffected controls (representative examples). These mutations were not present in the controls or other HLHS samples.

Since the dysfunction of HLHS patient specific cardiomyocytes seems to be conserved across a number of patients and develops *in vitro*, independently of the growth of other structures that contribute to the formation of the embryonic heart, a genetic contribution to HLHS manifesting in cardiomyocytes seems more likely and we therefore sought to identify genetic variants that could predispose to HLHS. We performed whole exome sequencing on the five HLHS patients using the Agilent SureSelect system exome capture and sequencing protocol. Despite the small sample size and lack of parental or sibling DNA (this preventing identification of mode of inheritance), we sought to identify deleterious variants that may elucidate potential and common mechanisms for the cellular phenotypes described above. Deleterious in our study was defined by at least one of the predictors [SIFT (32) or PolyPHEN2 (33)] and those that had alternative allele frequency less than 0.01 in both 1000 Genomes project and ESP6500 (Table 2). Over 200 deleterious variants were identified in each of the five HLHS patients. Further selection of these variants was carried out using the LifeMap Discovery Tools [VarElect, GeneCards, MalaCards (34,35)] and PubMed using the search terms: cardiomyocytes, HLHS, mitral stenosis, aortic stenosis, bicuspid valve, outflow tract problems, cardiac vasculogenesis, patent ductus arteriosus, congenital heart disease, heart development, cardiomyocyte and smooth muscle development and sarcomere assembly and function. An important final criterion for our analysis was communality of deleterious variants between all HLHS patients. From this analysis, we observed that three of the HLHS patients (HLHS3, HLHS6 and HLHS) harboured at least one deleterious variant in NOTCH signaling receptors (*NOTCH1, NOTCH3* and *NOTCH4*) genes (Table 3) despite these variants not being identical in each patient. The remaining two patients (HLHS1 and HLHS2) harboured variants in NOTCH receptor genes (*NOTCH4* and *NOTCH2*), although the later were not predicted to be deleterious from our bioinformatic analysis. Furthermore, all HLHS patients harboured deleterious variants in multiple genes involved in the NOTCH signaling pathway (Table 3). In total 8 variants in *NOTCH* receptors were found in HLHS fibroblast samples, four of which were previously reported (11,36,37), with the remaining four being novel (*NOTCH3*:exon11:c.A1766C:p.Q589P, *NOTCH4*: exon1:c.33_44del:p.11_15del, *NOTCH4*:exon1:c.17_28del:p.6_10del and *NOTCH4*:exon18:c.G2834A:p.C945Y (Fig. 5A). The presence of all 8 variants was confirmed by direct sequencing (Fig. 5B). These variant loci span a wide range in *NOTCH* receptor gene open reading frames involving signal peptide, EGF-like repeat domains, transmembrane region and transcriptional activation domain which might interrupt NOTCH trafficking, receptor activation, proteolytic cleavage cascade and transcriptional activating functions. Together, these data suggest that mutations in key components of the NOTCH signalling pathway leading to dysfunction of this important pathway may underline the HLHS aetiology.

**Table 2:** Summary of exome sequencing performed on dermal skin fibroblasts obtained from the HLHS patients. A filtering strategy was used for each patient as follows:

1. Exonic variants were generated by removing Synonymous SNVs and non-exonic variants.
2. Rare exonic variants were generated by removing variants with alternative allele frequency in either 1000Genomes project/ESP6500/cg69/inHouseExomes/InHouse GATK Exomes greater than 0.05.
3. Rare interested hits were generated if they had both 1000Genomes Freq < = 0.01 and ESP6500 Freq < = 0.01.
4. X linked variants were generated by removing variants that were not on chromosome X.
5. Homozygous variants were generated by removing variants that were not on autosomal chromosomes, compound heterozygotes and heterozygous variants.
6. Compound heterozygotes were generated by keeping genes with two or more rare variants.
7. Deleterious variants were generated by selecting variants that were predicted Deleterious by at least one of the predictors (SIFT or PolypHEN2) and those that had alternative allele frequency less than 0.01 in both 1000Genomes project and ESP6500.

**Table 3:** Summary of variants identified by exome sequencing analysis in genes involved in the NOTCH signalling pathway. Deleterious variants are shown in bold and compound heterozygotes have been underlined

	HLHS	HLHS1	HLHS2	HLHS3	HLHS6
**Exanic variants**	35968	33891	35510	34651	35834
**Rare exonic variants**	2866	2610	2974	2659	2841
**X-linked**	42	19	82	24	74
**Homozygous**	125	108	127	121	140
**Compound heterozygotes**	514	517	711	524	579
**Deleterious hits**	281	238	272	265	288

**Figure 6:**
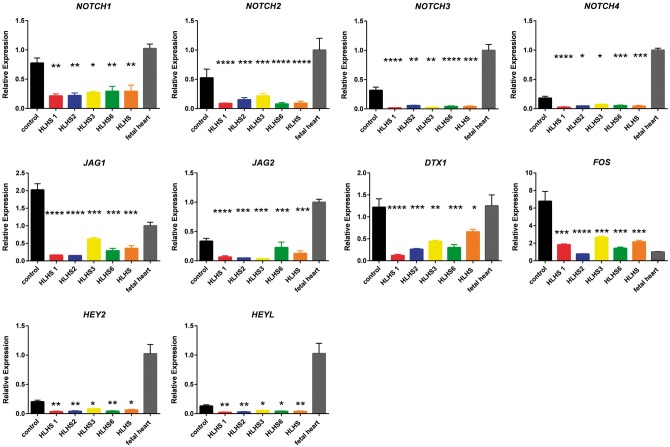
Reduced expression of key components of NOTCH signalling pathway in HLHS iPSC lines. Quantitative RT-PCR analysis performed on day 14 iPSC- derived cardiomyocytes showing decreased expression of NOTCH receptors, NOTCH ligands and targets in HLHS-iPSC-derived cardiomyocytes when compared to unaffected controls. Data are presented as mean+/- SEM. The values for fetal heart sample were set to 1 and all other values were normalised against this. One way Anova analysis with Dunnett multiple comparison tests was performed. ****P < 0.0001; ***P < 0.001; ** *P* value between 0.001 and 0.01 *P value between 0.01 and 0.05. HLHS iPSC lines: *N =* 6 (2 clones × triplicate biological repeats), control iPSC lines: *N =* 12 (2 clones × triplicate biological repeats × 2 unaffected control iPSC lines).

**Figure 7:**
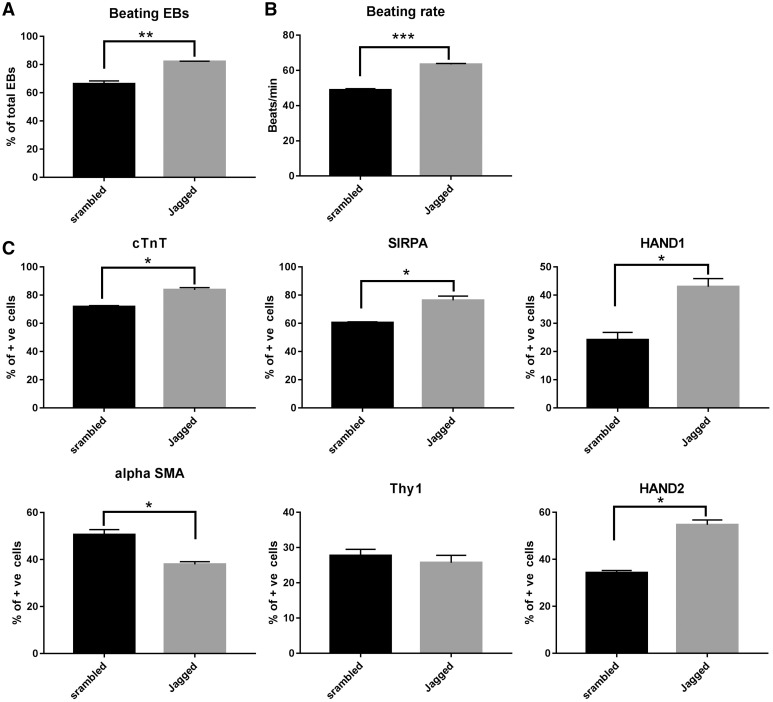
Activation of NOTCH signalling pathway restores the ability of HLHS- iPSC lines to give rise to cardiomyocytes. (**A**) Analysis of the percentage of EBs that generate contraction during differentiation of Jagged and scrambled peptide treated HLHS-iPSC line; (**B**) Analysis of beating frequency of EBs that generate contraction during differentiation of Jagged and scrambled peptide treated HLHS-iPSC line; (**C**) Flow cytometry data analysis demonstrating that Jagged treatment enhances the ability of HLHS-iPSC to give rise to cardiomyocytes and reduces the ability to differentiate to smooth muscle cells when compared to scrambled peptide treated control cells at day 14 of the differentiation time-course. T-test analysis was carried out, ***P < 0.001; **P value between 0.001 and 0.01; *P value between 0.01 and 0.05. Data are presented as mean+/- SEM, *n =* 3+.

To validate the dysfunction of NOTCH signalling pathway in HLHS-iPSC-derived cardiomyocytes, we performed quantitative RT-PCR analysis which indicated a significant downregulation in the expression of *NOTCH1-4* receptors and NOTCH targets (*DTX1, FOS, HEY2* and *HEYL*), thus confirming the impairment of NOTCH signaling pathway in HLHS-iPSC-derived cardiomyocytes (Fig. 6). It is of interest to note that we also observed downregulation of NOTCH-binding proteins (*JAG1* and *JAG2*) which may reflect an autocrine feedback loop in NOTCH signalling pathway. Downregulation of several NOTCH targets and NOTCH binding proteins including *HEY2* and *JAG2* was also confirmed by the RNA-seq analysis described in previous results section (data not shown). To further assess the role of dysfunctional NOTCH signalling on the aetiology of HLHS, we performed HLHS-iPSC differentiation to cardiomyocytes in the presence of a Notch ligand (Jagged peptide) or Scrambled control as described by Arumugam *et al.* (38). The addition of Jagged peptide was carried out in HLHS1 patient which has two *NOTCH4* mutations in the protein signal peptide domain (Fig. 5 and Table 3). Mutations in the signal peptide domain are thought to affect the cytoplasmic expression of Notch proteins and inhibition of proteasome activity, thus impacting on NOTCH protein processing and function (39). However, such mutations are unlikely to affect Jagged binding to Notch4 and activation of Notch pathway in cardiomyocytes derived from this patient. Furthermore the other Notch ligands (NOTCH1, NOTCH2 and NOTCH3) can also be activated by Jagged 1 addition, thus resulting in an overall increase in the activity of NOTCH signalling which can rescue the impact of NOTCH4 mutation in these cells. Activation of Notch signalling was confirmed by increased expression of Notch targets such as *DTX1, FOS, HEY1, HEY2* and *HES1* (Supplementary Material, Fig. S6). This led to a significant increase in capacity to generate cardiomyocyte clusters and beating rate (Fig. 7A and B) as well as increased percentage of cells expressing HAND1 and HAND2 by day 14 (Fig. 7C) as well as cardiomyocyte specific markers cTnT and SIRPA at both days 14 (Fig. 7C) and 21 of differentiation (data not shown). Furthermore, the percentage of cells expressing the smooth muscle markers (α-SMA) was significantly decreased at both days 14 (Fig. 7C) and 21 (data not shown), suggesting a restoration of cardiomyocyte differentiation capacity in HLHS-iPSC lines. Together, these data indicate that dysfunction of NOTCH signalling pathway is at the core of HLHS aetiology.

## Discussion

In this current study, we have highlighted dysfunctional Notch signalling in the aetiology of HLHS. In a previous publication, we reported the successful derivation of iPSC from a single HLHS patient and differentiation towards cardiomyocytes (28). Although we were able to show a range of cellular, functional and structural deficiencies in HLHS derived cardiomyocytes, it was unclear whether these were common features underlined the HLHS aetiology. In this present study, we report the derivation of iPSC lines from an additional four HLHS patients and provide evidence that HLHS-iPSC-derived cardiomyocytes are characterised by the following common deficiencies: (1) reduced beating rate, (2) disorganised sarcomere structure and (3) sarcoplasmic reticulum dysfunction, all of which are reflected at the gene expression level as revealed by RNA-seq analysis. These data replicate the initial findings published by our group (28) and show that the multiple expression, structural and functional differences are reproducible *in vitro* characteristics of iPSC lines derived from patients with HLHS compared to controls which implies that patient cardiomyocyte-specific factors make a substantial contribution to the development of HLHS. Our data also indicate that cardiomyocyte specific defects are not an isolated event and are associated with deficiencies observed during the iPSC differentiation process which include a significant reduction in the ability of HLHS-iPSC to give rise to beating cardiomyocyte clusters and reduced expression of mesodermal, cardiac progenitors and cardiomyocytes markers reported by our group and others previously (12,17,28). This reduced emergence of cardiomyocytes was compensated for an increased presence of cells expressing the typical smooth muscle markers, which suggests an altered cell fate decision during early development with respect to cardiomyocyte or smooth muscle formation. Smooth muscle is very different from cardiac muscle in many aspects including function, structure, contraction and excitation-contraction coupling. It is possible that replacement of cardiomyocytes with smooth muscle cells would contribute to reduction of blood flow through the left ventricle which seems to be a key factor for the development of HLHS in some patients.

To date, increasing evidence supports a genetic basis for HLHS and these include observations of familial clustering and concurrence with specific chromosomal disorders (6,25) as well as the presence of deleterious variants in genes such as *NOTCH1, NKX2.5*, *GJA1* and more recently *MYH6* (40). The multiplicity of common cellular deficiencies observed during differentiation of all HLHS-iPSC lines to cardiac lineages led us to speculate that mutations in the same gene or genes playing a role in a common pathway(s) may be responsible for HLHS phenotypes uncovered by the iPSC disease model. Our whole exome sequencing analysis revealed the presence of deleterious variants in *NOTCH* receptor genes or other genes involved in the NOTCH signalling pathway. Two out of the five HLHS patients (HLHS3 and HLHS) harboured two and four variants in *NOTCH1* and *NOTCH4* genes, respectively; one patient (HLHS1) harboured two different variants in *NOTCH4* gene and the remaining two patients (HLHS2 and HLHS6) harboured single variants in the *NOTCH2* and *NOTCH3* genes. All patients also harboured deleterious variants on other genes implicated in NOTCH signalling which led us to hypothesise that dysfunction of NOTCH signalling was the key underlining mechanism for HLHS. While *NOTCH1* mutations have been linked with HLHS (12,17), the presence of *NOTCH2, NOTCH3* and *NOTCH4* variants is novel and not associated with the disease previously. We were unable to collect DNA from the families of HLHS patients to assess whether these were *de novo* or inherited changes. Nonetheless, we were able to demonstrate that HLHS-iPSC-derived cardiomyocytes had lower expression of all four *NOTCH* receptor and target genes which indicates impaired NOTCH signalling activity in these cells. Most importantly, activation of NOTCH signalling pathway via addition of Jagged ligand, resulted in reversal of the key cellular and functional phenotypes (e.g. restoration of beating rate, emergence of beating clusters, enhanced generation of cardiomyocytes and suppression of smooth muscle formation) in HLHS-iPSC-derived cardiomyocytes which strongly suggests that the dysfunction of the NOTCH signalling pathway lies at the heart of the HLHS aetiology.

Notch signalling is an evolutionary conserved pathway with important functions in many cellular events, including cellular communication, maintenance of tissue boundaries, cell fate acquisition, stem cell renewal and differentiation. Despite a wealth of literature already published for Notch signalling, contradictory reports exist on its role in differentiation of pluripotent stem cells to cardiac lineages. Schroeder and colleagues suggested that Notch signalling plays various roles during the differentiation process and that its activation in murine models results in suppression of mesodermal cell generation and their further differentiation to cardiac muscle (41). Contrary to these reported findings, a current report, suggests that biphasic Notch regulation is needed for differentiation of murine embryonic stem cells (mESC) and iPSC, since activation of Notch signalling favours mesodermal induction at the early stages of differentiation; however this is deleterious for formation of mature cardiac progenitors at the later stages of differentiation (42). Activation of Notch 1 or Notch 4 signalling in mESC derived haemangioblasts respecifies them to a cardiac fate (43), further adding to the evidence that at least in the mouse pluripotent stem cell model, Notch signalling plays different roles depending on the temporal differentiation window and specific target cells.

In the human embryonic stem cell (hESC) system, it has been reported that NOTCH inhibition promotes differentiation of cardiac mesoderm, whilst NOTCH activation leads to an increase in neural and smooth muscle cell markers (44,45); however, these studies did not apply a biphasic strategy of NOTCH inhibition as in mouse, leaving open the question of whether persistent or phase-specific NOTCH inhibition is beneficial to hESC differentiation to cardiac lineages. However, in human circulating progenitor cells, expression of α-sarcomeric actinin and cTnT is associated with NOTCH signalling and is blocked by preventing Notch signals with Y-secretase inhibitors (46), which suggest that similarly to mouse cardiac progenitors, activation of NOTCH signalling is essential for the acquisition of a cardiac progenitor fate in humans. The HLHS-iPSC model we have described herein provides an excellent model system from which we can infer new insights on the role of Notch signalling during iPSC differentiation. Our data suggest that a reduced NOTCH signalling affects all stages of differentiation starting from commitment to mesoderm, emergence of beating clusters, beating rate and their further differentiation to cardiomyocytes. Furthermore, a lower NOTCH signalling activity seems to shift the differentiation balance from cardiomyocytes to smooth muscle cells and such knowledge can be used to manipulate the differentiation outcomes by manipulating NOTCH activity with small molecule inhibitors or activators.

Currently, is not known, how Notch signalling may affect the sarcoplasmic reticulum dysfunction observed in cardiomyocytes derived from these HLHS patients. A recent report has suggested that activation of Notch signaling by short term treatment with Jagged-1 enhances store-operated calcium entry in human pulmonary arterial smooth muscle cells in a dose dependent manner (47). Hence it is possible, that activation of Notch signalling may restore calcium homeostasis in HLHS-iPSC-derived cardiomyocytes; however this needs to be investigated further. It is also possible that mutations in genes other than Notch receptors are responsible for the sarcoplasmic reticulum dysfunction. For example, variants in Calcium Voltage-Gated Channel Auxiliary Subunits (*CACNB2, CACNA1I, CACNA1C etc.)* were identified in several of the HLHS patients included in this study. The significance of these findings need to be validated by further molecular and functional studies and is currently ongoing in our group.

In summary, we have established a reliable model of HLHS using the iPSC disease modelling approach and have revealed consistent cellular and functional differences between unaffected HLHS iPSC-derived cardiomyocytes which are underlined by likely mutations in genes involved in the NOTCH signalling pathway. Although HLHS is an uncommon condition, and multiplex HLHS families are rare, investigation of the biological mechanisms leading to HLHS could have wide importance. Understanding cardiomyocyte growth and differentiation has become a major research priority since the development of hESC and iPSC technologies in the hope that these might contribute to cardiac repair strategies. In this manuscript, we have revealed an important role for a key signalling pathway (NOTCH) and its role during pluripotent stem cell differentiation, thus providing important insights into early human cardiomyocyte development and potential tools and strategies for enhancing stem cell differentiation to desired lineages (cardiomyocytes versus smooth muscle commitment). Furthermore, our data have provided novel genetic insights which can be taken forward for establishing molecular diagnosis tests for HLHS patients and genetic counselling of affected families.

Last but not least, high throughput screening to identify additional compounds that restore the genetic and functional profile of HLHS cardiomyocytes to normal could result in novel therapies, in the setting of *in vitro* growth promotion of cells for cardiac regenerative medicine.

## Materials and Methods

### iPSC generation

Dermal fibroblasts were obtained from skin biopsies of all five HLHS patients characterized by mitral and aortic atresia (Table 1). iPSC derivation was performed using a CytoTune®-iPS 2.0 Sendai Reprogramming Kit (A16517) from Thermo Fisher Scientific (Waltham, MA). In brief, fibroblasts were transduced with the reprogramming vectors KOS (a polycistronic vector encoding *KLF4*, *OCT4, SOX2*), h*c-Myc* and h*Klf4* following manufacturer’s instructions. One week after transduction, fibroblasts were disaggregated and plated onto feeder layers of mitotically inactivated mouse embryonic fibroblasts in hESC culture medium (KO-DMEM, 20% Knockout™ Serum Replacement, 0.1 mMol nonessential amino acids, 2 mMol L-glutamine, 100 units/ml penicillin and 8ng/ml human recombinant bFGF, all from Thermo Fisher Scientific) at a density of 8,000 cells per well of a six well plate. The feeder plates with Sendai vector treated cells were maintained at 37 °C and 5% CO_2_ in hESC medium for 2-3 weeks or until colonies with typical hESC morphology appeared. Individual colonies were mechanically dissected and plated onto Matrigel-coated plates (Corning, NY) using mTeSR media (Stem Cell Technologies, Vancouver, British Columbia, Canada) medium.

### iPSC differentiation to cardiomyocytes

Differentiation of iPSCs towards cardiomyocytes was achieved using a modified Keller protocol with StemPro-34 SFM medium (Thermo Fisher Scientific) supplemented by growth factors (30). iPSC colonies were dissociated and detached by enzymatic treatment with 1mg/ml collagenase IV and 0.5 mg/ml Dispase (Thermo Fisher Scientific) to form embryoid bodies (EBs) in suspension culture with ultra-low attachment plates (Corning). A summary of the cardiac differentiation protocol together with concentrations and times of application of growth factors and signalling molecules is shown in Figure 2A. BMP4, Activin A, VEGF, DKK1 and SB431542 were purchased from R & D technologies (Minneapolis, MN), bFGF from Thermo Fisher Scientific, Dorsomorphin from Sigma (St. Louis, MO) and Y27632 from Chemdea (Ridgewood, NJ). At day 7, the EBs were seeded onto Matrigel-coated plated and further cultured up to 30 days in StemPro-34 SFM media supplemented with VEGF and bFGF. To observe the effect of Notch signalling activation on cardiomyocyte induction from iPSCs, 10 μM of Jagged (Jag-1 DSL peptide, ANA61298, AnaSpec, Fremont, CA) was added into medium from day 3 of differentiation. As control, 10 μM scrambled peptide (ANA64239, AnaSpec, Fremont, CA) was applied in parallel. At day 14 of differentiation, quantitative RT-PCR was carried out to examine the gene expression levels of Notch ligands, receptors and targets.

### Immunocytochemistry

iPSC-derived cardiomyocytes were fixed in situ with 4% paraformaldehyde (PFA) fixative for 30 min. After 3 washes with PBS, cells were permeabilised in 0.2% Triton X-100 for 30 min, blocked for 1 h in 10% FCS and 1% BSA in phosphate buffer saline (PBS), and incubated overnight at 4 °C with primary antibodies diluted 1:200 in 1% BSA in PBS. The second day, the cells were washed 3 times with PBS and incubated with fluorescence-conjugated secondary antibodies and DAPI in 1% BSA in PBS for 1 h at room temperature. Images were taken by Eclipse Ti Confocal Microscope (Nikon, Minato-ku, Tokyo). The antibodies included anti-β-III-tubulin (Thermo Fisher Scientific, A25532), anti-AFP (Thermo Fisher Scientific, A25530), anti-SMA (Thermo Fisher Scientific, A25531), anti-cTnT (Abcam, Cambridge, MA, Ab64623), anti-α-Actinin (Thermo Fisher Scientific, MA1-22863).

### Flow cytometric analysis

iPSCs and cardiomyocytes derived therefrom were dissociated by incubation with Accutase (Thermo Fisher Scientific) for 5 min at 37 °C. A total of 1×10^6^ single cells were resuspended in PBS supplemented with 5% fetal calf serum (FCS) and stained for the presence of appropriate markers. For intracellular proteins, staining was carried out on cells fixed with 4% paraformaldehyde for 10 min at 37 °C and permeabilised with cold methanol at -20 °C. The primary antibodies included TRA-1-60 (1:200) (Millipore, Ontario, Canada, FCMAB115F), NANOG (1:50) (Cell Signaling Technology, Danvers, MA, 5448s), BRACHYURY (1:20) (R&D, IC2085P), ISL1 (1:20) (Abcam, ab20670), NKX2.5 (1:100) (R&D, MAB2444), cTnT (1:100) (Thermo, MS-295-P), SIRPA (1:20) (Biogelend, 323808), HAND1 (1:50) (Abcam, Ab46822), HAND2 (1:50) (Abcam, Ab56590), αSMA (1:20) (Abcam, Ab32575), Thy1 (1:20) (BD, 559869), Connexin-43 (1:20) (Sigma, C 6219). At least 10,000 cells were analyzed for each experiment on an LSRII flow cytometer and data were analyzed with FACSDiva software (BD Biosciences, Franklin Lakes, NJ).

### Teratoma formation in severe combined immunodeficient mice

All procedures involving mice were carried out in accordance with institutional guidelines and permission. Approximately 1×10^6^ iPS cells were injected subcutaneously into the right flanks of adult NOD/SCID mice. All cells were co-transplanted with 50µl Matrigel (BD Biosciences) to enhance teratoma formation. After 70–90 days, mice were sacrificed and teratoma tissues were dissected. Material for histological analysis was fixed in Bouins fixative [70% saturated picric acid (Sigma); 25% formaldehyde (37%/40%, Sigma); 5% glacial acetic acid (Sigma)] overnight. Tissues were processed, sectioned to 6μm according to standard procedures and counterstained with either Haematoxylin and Eosin or Massons trichrome stain. Sections were examined using bright field microscopy and photographed as appropriate.

### Exome sequencing

DNA was extracted from 5 HLHS patient fibroblasts using QIAamp DNA Micro kit (Qiagen, Germantown, MD, 56304). Exome sequencing was performed by BGI. Exome capture was performed using Agilent SureSelect Human All Exon kit (V4). Libraries were constructed following the Illumina Paired-End Sequencing Library Preparation Protocol version 1.0.1 and then sequenced on the Illumina GAIIx platform with version 4 chemistry and version 4 flowcells. The sequencing reads were analyzed using the following workflow to identify variants in patient. The quality of sequencing reads was firstly checked with FastQC (Version 0.11.2) (48). Low quality bases (Q < 20) on 3’ ends of reads were trimmed off using seqtk (Version 1.0) (49). Duplicated reads were then removed with FastUniq (Version 1.1) (50) before mapping to the human reference genome GRCh37 with BWA (Version 0.7.6.a) (49). The alignments were refined with tools of the GATK suite (Version 3.2) (51). Variants were called according to GATK Best Practice recommendations, (52,53) including recalibration. Freebayes (Version 1.0.1) (54) was also used to call variants from the same set of samples. The variants called by Freebayes with total coverage ≥ 5, minor allele coverage ≥ 5 and variants call quality ≥ 20 were added to those identified by GATK. Non-synonymous exonic variants were subsequently filtered by quality and minor-allele frequency (MAF) reported in the 1000 Genomes project (2012 Feb release) (55) and ESP6500 (56). Variants with MAF > 0.05 in either of the databases were excluded. ANNOVAR (Version 2014-07-22) (57) was used for annotations and prediction of functional consequences. The presence of candidate deleterious variants was confirmed by amplifying the regions of interest by PCR and subjecting those to direct sequencing. The DNA oligonucleotide sequences used are shown in Supplementary Material, Table S2.

### Transmission electron microscopy

Transmission electron microscopy was performed on day 30 contracting EBs fixed with 2% glutaraldehyde in 0.1M cacodylate buffer. Post fixation was performed at room temperature for 1 h with 1% osmium and 1.5% potassium ferrocyanide. The samples were dehydrated in graded acetone and embedded in epoxy resin at 60 °C. Half micron sections were stained with 1% toluidine blue and ultra-thin section were cut on Leica EM UC7 ultramicrotome and double stained with 1% uranyl acetate and lead citrate. Ultrastructural examination was performed with Philips CM 100 TEM at 100 kV. Digital images were recorded with an AMT40 CCD camera (Deben, Bury St. Edmunds, Suffolk, UK).

### Quantitative RT-PCR

Total RNA was isolated using ReliaPrep™ RNA Cell Miniprep System (Promega, Madison, MI) at different points during the differentiation process. 1µg of total RNA was used for reverse transcription using the GoScript™ Reverse Transcription System (Promega). Quantitative RT-PCR was performed using a Quant Studio 7 real-time PCR system with the GoTaq qPCR Master Mix (Promega). Data were analysed using the comparative threshold cycle (Ct) method. In all samples, the results were normalised to the expression level of the housekeeping gene *GAPDH*, and referenced to human embryonic heart cDNA (obtained from Human Developmental Biology Resource, Carnegie Stage 14). Primer sequences for qRT-PCR assays are shown in Supplementary Material, Table S3.

### Generation of iPSC-derived cardiomyocytes and electrophysiological recordings

To facilitate electrophysiological recordings and RNA-seq analysis described below, a monolayer based method was used for generation of cardiomyocytes. In brief, iPSCs were passaged at a density such that they reached confluence 3–4 days after passaging. On day 0 of differentiation, mTeSR1 was replaced with RPMI supplemented with 2% B27 without insulin (both from Life Technologies). On day 1 this basal medium was supplemented with CHIR99021 trihydrochloride (Tocris) at either 6, 9 or 12 µM concentrations depending on the cell line and passage. On day 1 the medium was changed to base medium. On day 3 the medium was changed to a mixture of fresh basal medium and the existing medium from the culture in a 1:1 ratio, this was supplemented with 5µM IWP2 (Tocris). On day 5 the medium was changed to base medium. On day 7 the medium was changed to maintenance medium consisting of RPMI supplemented with 2% B27. On day 9 the medium was changed to metabolic purification medium consisting of RPMI without glucose supplemented with 2% B27. Purification medium was changed on days 11 and 13. On day 14 the medium was changed back to maintenance medium. On day 15 the medium was changed to maintenance medium supplemented with 10µM Y27632 and incubated for 2 h in standard conditions. The cultures were then washed with DPBS and incubated 0.25% trypsin/EDTA in standard conditions for 5–20 min until the fragments detached from the culture surface. Fragments were gently triturated to produce clusters of cells and equal volume of RPMI with 20% FBS added to inactivate the trypsin. The cell suspension was centrifuged at 200g for 4 min at 20 °C, the supernatant aspirated and the pellet re-suspended in RPMI with 20% FBS supplemented with 10 µM Y27632. The concentration of cells in suspension was estimated using a haemocytometer and the cells were transferred to matrigel coated culture-ware at a density of 0.125 – 0.25 ×10^6^ cells per cm^2^ and incubated overnight in standard conditions. The follow day the medium was changed to maintenance medium which was changed every other day thereafter.

On day 25-30 of differentiation cultures were re-plated using the same method of enzymatic dissociation into matrigel coated wells of a 96 well glass bottomed plate (MatTek, Ashland, MA) using the same technique. Between 40–80 ×10^3^ cells were plated in each well depending on the cell line. A fraction of the cells were used for the assessment of differentiation and purification efficiency by flow cytometry. Cells were fixed for 30 min in 4% formaldehyde at room temperature and then permeabilised with 0.1% Triton X-100 for 15 min. Cells were blocked by incubation with 10% FBS for 30 min. Aliquots of 1×10^5^ cells were incubated with antibodies to the proteins if interest in a volume of 100ul, in the dark, at room temperature for 45 min. The antibodies used were: anti-cTnI-Alexa647 (1:20) (BD Biosciences), and an isotype controls from the same manufacturer and matched for protein concentration. Samples were washed once using a BD wash/lyse prior to analysis with an LSRII (BD Biosciences) flow cytometer. Flow cytometry data was analysed using FACS Diva software (BD). After the exclusion of debris and doublets samples were excluded if <75% of single cell events were classified as positive for the anti-cTnI label.

On day 30–40 the medium was replaced 2 μM Fura-4F in serum free medium (SFM) and incubated for 30 min in standard conditions. SFM consisted of DMEM without phenol red supplemented with 10mM galactose, 10mM sodium pyruvate and 2 mM L-glutamine (all from ThermoFisher Scientific). Cells were washed with SFM and incubated for a further 30 min in SFM. Calcium transients were recorded using the CellOPTIQ® platform (Clyde Biosciences Ltd) (58). For each well of the plates, the Fura-4F signal was recorded from a 0.2 mm × 0.2 mm area using a 40× (NA 0.6) objective lens. Ratiometric imaging was performed using fast switching between light-emitting diode (LED) excitation wavelengths of 355 ± 10 nm and 380 ± 10nm. Emitted light was collected by a photomultiplier (PMT) at 510–560 nm. The two fluorescence signals were digitized at 1kHz, and the ratio of fluorescence (long wavelength/short wavelength) was used to identify the occurrence of calcium transients. Isoprenaline responses were determined by the addition of 1 µM isoprenaline (Sigma). Beat rates before and 5 min after isoprenaline application were determined by calculating the number of calcium transients in a 30 s period. For each cell line, at least 3 independent differentiation experiments were tested and cells from each differentiation experiment were plated into at least 3 wells of the 96 well plate. Control and disease lines were tested simultaneously with repeated analysis on 2 different dates. The mean beat rates and change in beat rates were calculated for cells from a given differentiation experiment and these means used for inferential statistics comparing cell lines.

### RNA-seq analysis (RNA-seq)

Total RNA was submitted to the Genomic Technologies Core Facility (GTCF). Quality and integrity of the RNA samples were assessed using a 2200 TapeStation (Agilent Technologies) and then libraries generated using the TruSeq® Stranded mRNA assay (Illumina, Inc.) according to the manufacturer’s protocol. Briefly, total RNA (0.1-4ug) was used as input material from which polyadenylated mRNA was purified using poly-T, oligo-attached, magnetic beads. The mRNA was then fragmented using divalent cations under elevated temperature and then reverse transcribed into first strand cDNA using random primers. Second strand cDNA was then synthesised using DNA polymerase I and RNase H. Following a single ′A′ base addition, adapters were ligated to the cDNA fragments, and the products then purified and enriched by PCR to create the final cDNA library. Adapter indices were used to multiplex libraries, which were pooled prior to cluster generation using a cBot instrument. The loaded flow-cell was then paired-end sequenced (76 + 76 cycles, plus indices) on an Illumina HiSeq4000 instrument. Finally, the output data was demultiplexed (allowing one mismatch) and BCL-to-Fastq conversion performed using Illumina’s bcl2fastq software, version 2.17.1.14. Reads from patient samples (88,023,772) and control (113,283,210 reads) were analysed with Kallisto (v0.43.0) (59) to quantify the abundances of transcripts and genes within each sample. Reads were pseudo-aligned to the human transcriptome GRCh38, release 79. Sleuth (60) (Wald test) was then used to calculate differential expression of transcripts using the Kallisto bootstrap estimates.

## Supplementary Material

Supplementary Figures

Supplementary Table 1

Supplementary Table 2

Supplementary Table 3
