# Supplementary material for: Induced pluripotent stem cell modelling of HLHS underlines the contribution of dysfunctional NOTCH signalling to impaired cardiogenesis
Source: Hum Mol Genet. 2017 May 17;26(16):3031–45. doi: 10.1093/hmg/ddx140 (PMC5886295; doi:10.1093/hmg/ddx140)
Supplement: Supplementary Figures [file suppl_figures_combined_ddx140.docx]

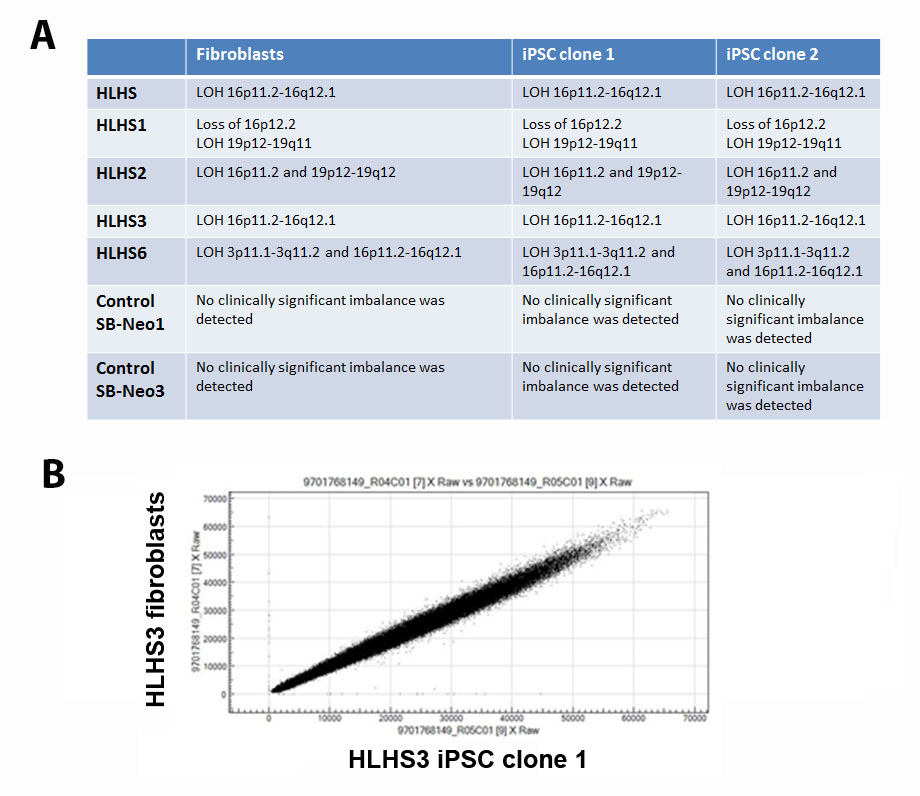


**Suppl. Figure 1. Genomic analysis of HLHS and control iPSC. (A)** Summary of karyotype data from CytoSNP analysis performed using the Illumina HumanCytoSNP-12 v2.1, LOH= loss of heterozygosity; **(B)** Representative example showing identical DNA profiles between parent fibroblasts and corresponding iPSC clone. A representative example is shown below for the HLHS3 samples.


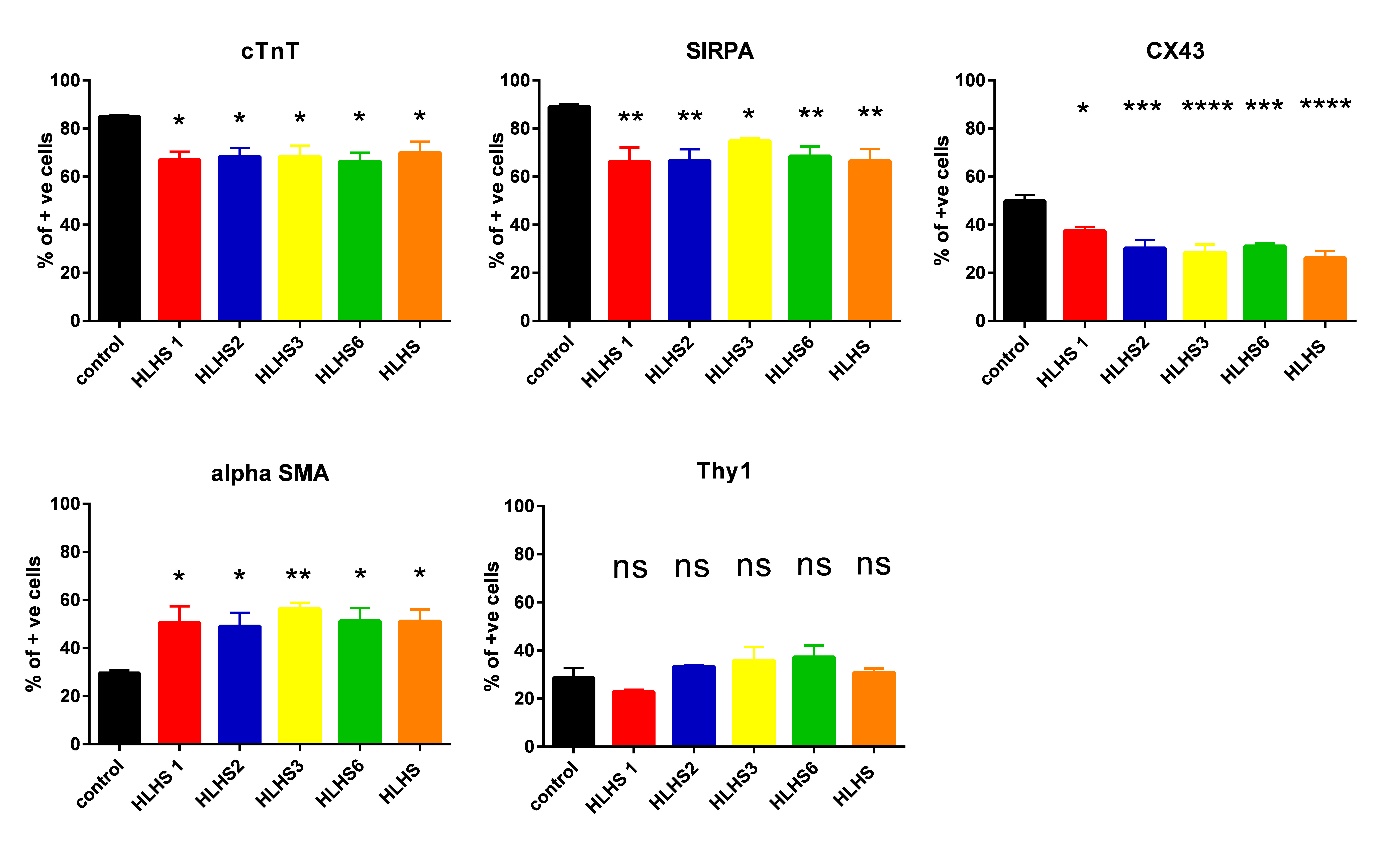


**Suppl. Figure 2. HLHS iPSC lines show an impaired ability to give rise to cardiomyocytes**. Flow cytometry data analysis demonstrating that HLHS-iPSC lines have a lower ability to give rise to cardiomyocytes but have an enhanced ability to differentiate to smooth muscle cells when compared to control derived cells at day 21 of the differentiation time-course. Anova analysis with Dunnett multiple comparison test was carried out, **** p < 0.0001; *** p < 0.001; ** p value between 0.001 and 0.01; * p value between 0.01 and 0.05. HLHS iPSC lines: N=6 (2 clones × triplicate biological repeats), control iPSC lines: N=12 (2 clones × triplicate biological repeats x 2 unaffected controls).


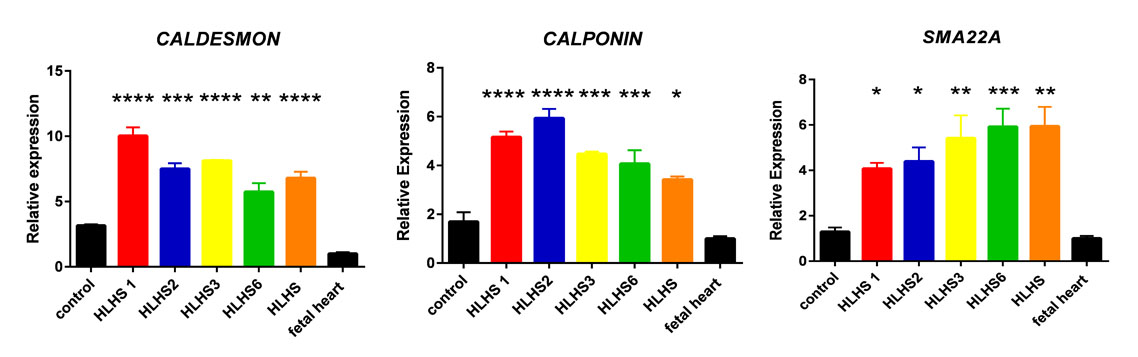


**Supplemental figure 3. Increased expression of smooth muscle cell markers in HLHS iPSC lines.** Quantitative RT-PCR analysis performed on day 14 iPSC- derived cardiomyocytes showing increased expression of smooth muscle markers in HLHS-iPSC derived cardiomyocytes when compared to unaffected controls. Data is presented as mean+/- SEM. The values for fetal heart sample were set to 1 and all other values were normalised against this. Anova analysis with Dunnett multiple comparison test was carried out, **** p < 0.0001; *** p < 0.001; ** p value between 0.001 and 0.01 * p value between 0.01 and 0.05. HLHS iPSC lines: N=6 (2 clones × triplicate biological repeats), control iPSC lines: N=12 (2 clones × triplicate biological repeats x 2 unaffected controls).


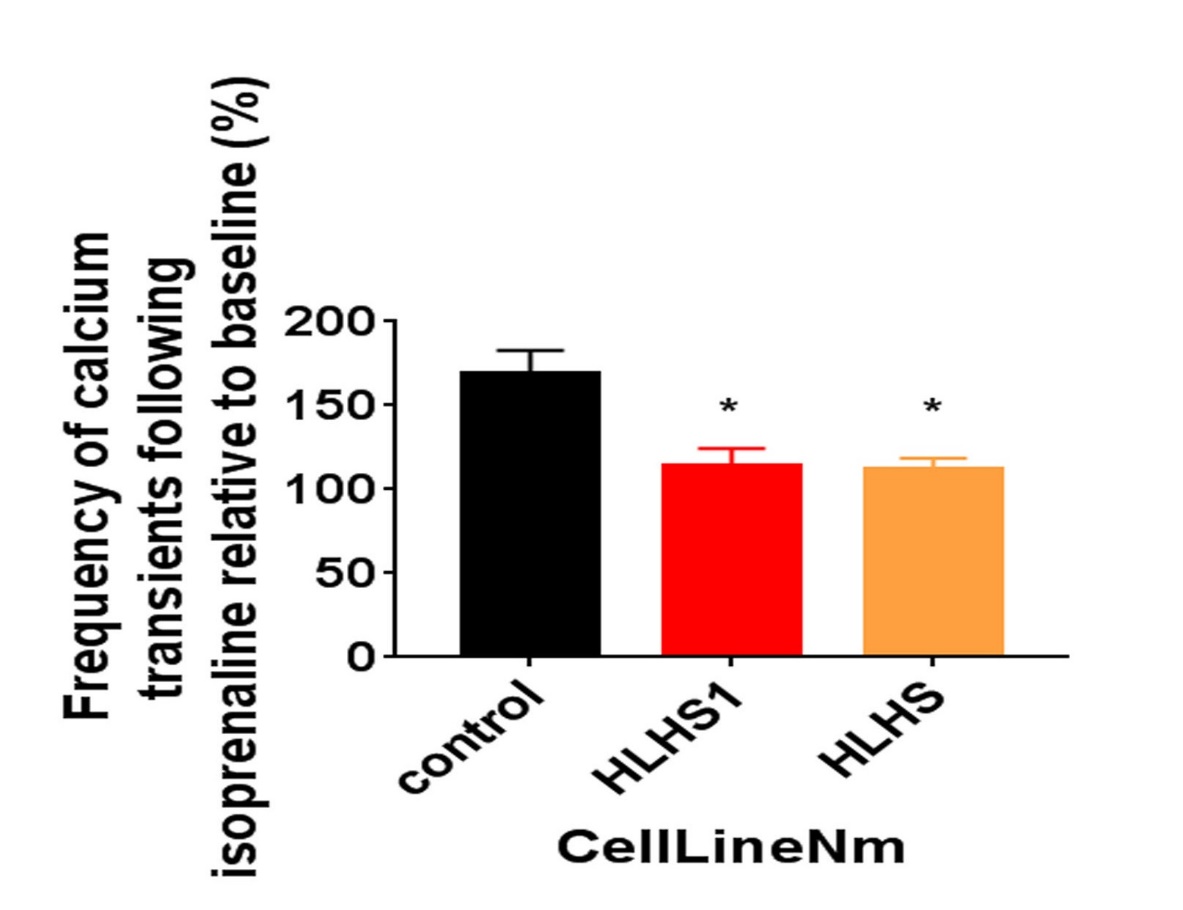


**Suppl. Figure 4. Measurement of Ca transients in iPSC derived cardiomyocytes following exposure to isoprenaline.** There was no significant difference in the baseline frequency of calcium transients between iPSC-CMs derived from the control line, HLHS1 and HLHS-iPSC lines (27±1 vs 36±6 vs 45±14 bpm). The frequency of calcium transients following isoprenaline exposure was calculated as a percentage of the baseline frequency. The mean frequency of transients after isoprenaline was less in HLHS1 and HLHS-iPSC lines derived cardiomyocytes when compared with control iPSC (115%±10 vs 113%±7 vs 170%±14, p<0.05 for each control vs disease comparison). Data are presented as mean+/- SEM. Continuous variables were compared using a one way ANOVA test with a Gabriel post-hoc test for comparisons between groups, and an α<0.05. The Shapiro-Wilk test p value and Levine’s test for equality of variance p value was <0.05 for all data-sets.


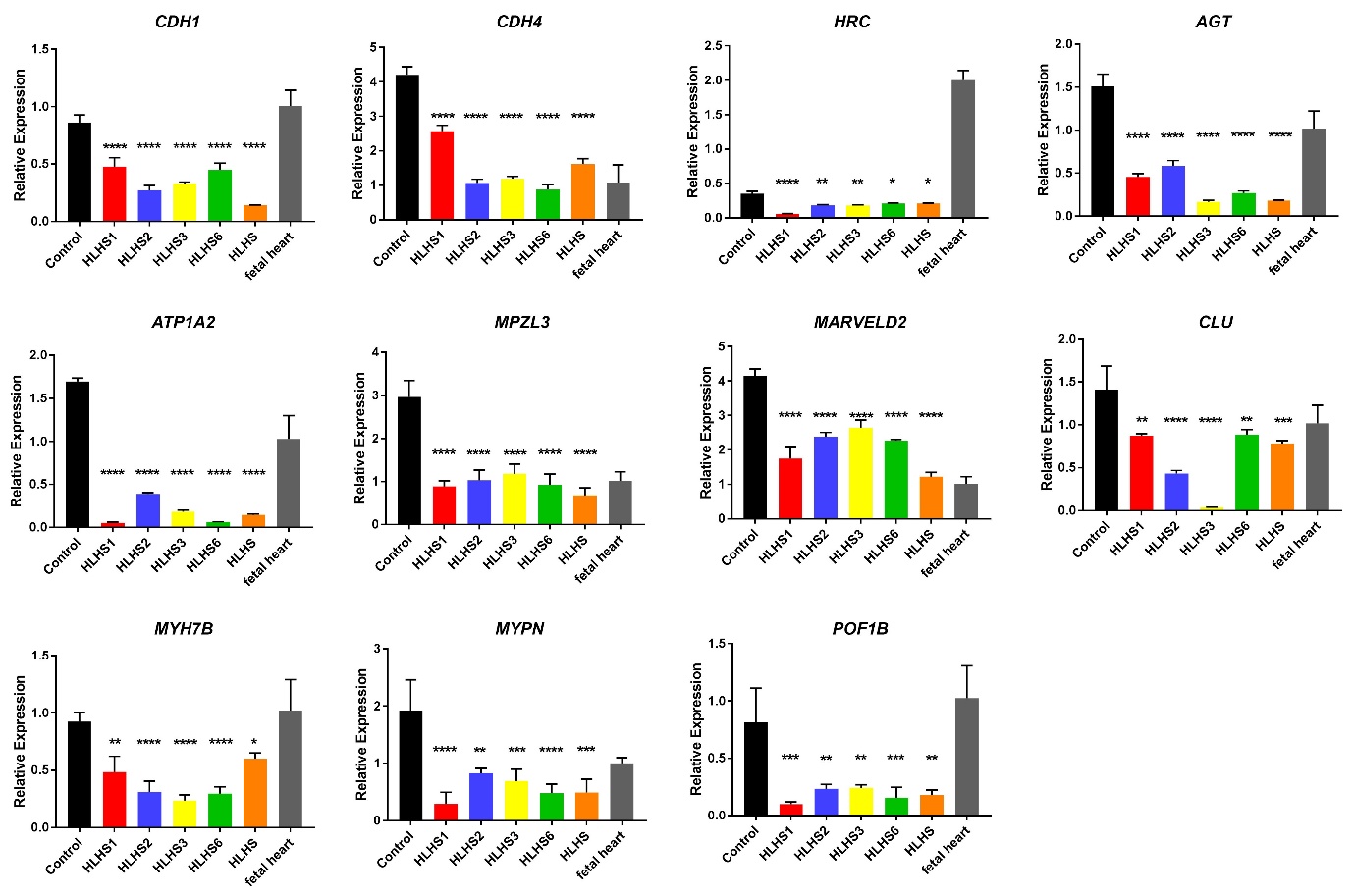


**Suppl. Figure 5. Decreased expression of genes related to cell-cell junction (*CDH1, CDH4*), regulation of heart rate (*HRC, AGT*), muscle contraction (*ATP1A2*), myocardial fibre morphology (*MPZL3, MARVELD2, CLU*) and Actin-binding proteins (*MYH7B, MYPN, POF1B*) in HLHS iPSC-derived cardiomyocytes.** Quantitative RT-PCR analysis performed on day 14 iPSC-derived cardiomyocytes showing decreased expression of above genes when compared to unaffected controls. Data is presented as mean+/- SEM. The values for fetal heart sample were set to 1 and all other values were normalised against this. Anova analysis with Dunnett multiple comparison test was carried out, **** p < 0.0001; *** p < 0.001; ** p value between 0.001 and 0.01 * p value between 0.01 and 0.05. HLHS iPSC lines: N=6 (2 clones × triplicate biological repeats), control iPSC lines: N=12 (2 clones × triplicate biological repeats x 2 unaffected controls).


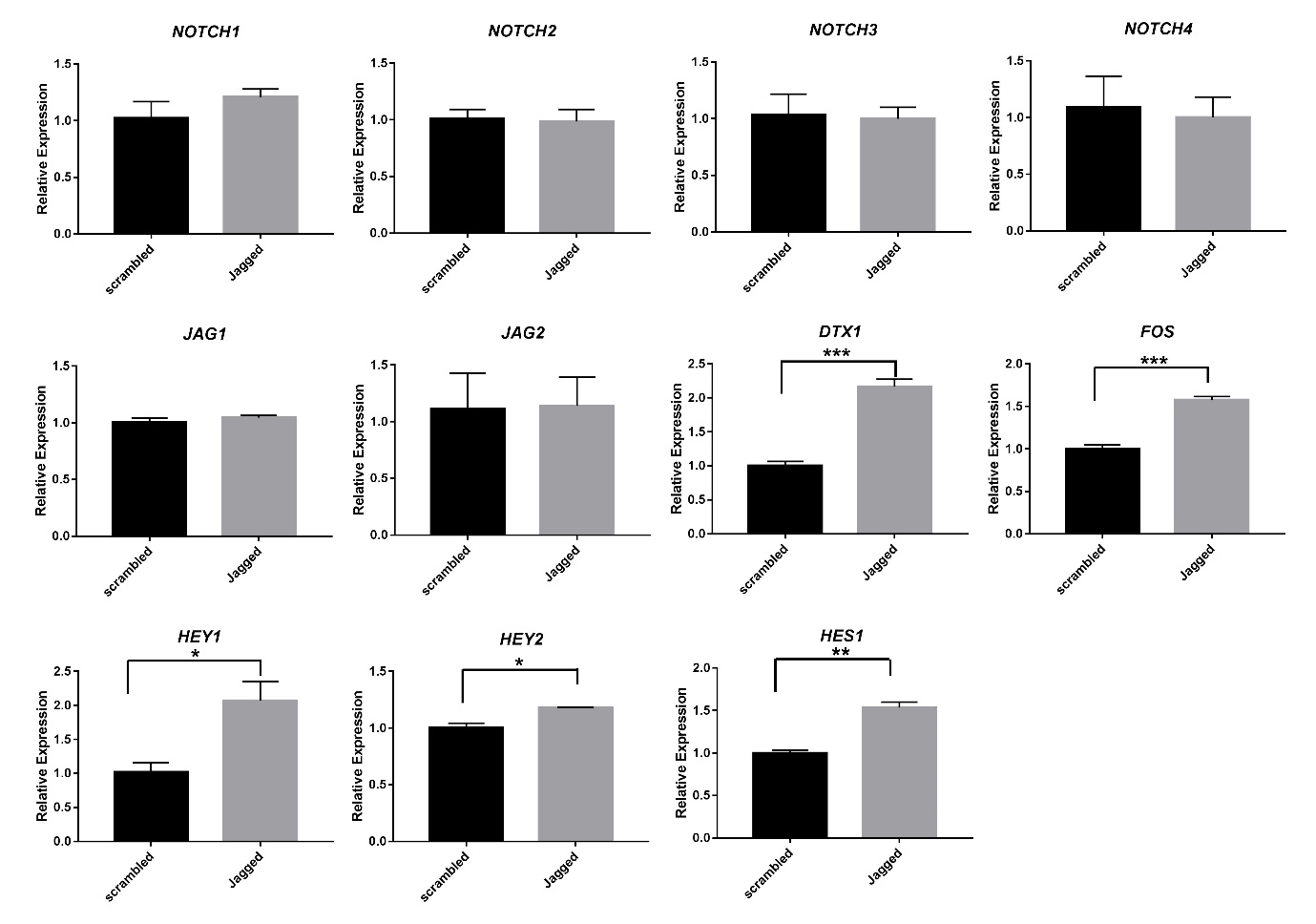


**Suppl. Figure 6. Activation of NOTCH signalling pathway via addition of Jagged ligand results in increased expression of *NOTCH* target genes.** Quantitative RT-PCR analysis of gene expression level of NOTCH receptors, NOTCH ligands and targets with day 14 iPSC- derived cardiomyocytes treated with Notch ligand Jagged peptide or scrambled control. Data is presented as mean+/- SEM, n=3. The values for scrambled control sample were set to 1 and Jagged group values were normalised against this. T-test analysis was carried out, *** p < 0.001; ** p value between 0.001 and 0.01; * p value between 0.01 and 0.05.

**Suppl. Table 1**. A list of significantly downregulated gene in day 21 cardiomyocytes derived from the HLHS sample compared to equivalent cells from SB-Neo3 control together with Go biological and molecular analysis. The columns in the table are:

- target_id – transcript or gene name

- pval - pvalue

- qval - FDR adjusted pvalue using benjamini-hochberg

-log fold change- log2 fold change

- mean_obs - the mean of the observations, this is used for the smoothing

- var_obs - the variance of the observations

- rss – residual sum of squares – estimates the model fit compared to ‘null’ model

- tech_var - the technical variance from the bootstraps

- sigma_sq - the raw estimator of the variance once the tech_var has been removed

- smooth_sigma_sq - the smooth regression fit for the shrinkage estimation

- final_sigma_sq - max(sigma_sq, smooth_sigma_sq). this is the one used for covariance estimation of beta (in addition to tech_var)

**Suppl. Table 2**. A list of DNA oligonucleotides used for confirming the presence of deleterious variants in *NOTCH* receptors in HLHS patients.

**Suppl. Table 3**. A list of DNA oligonucleotides used for the quantitative RT-PCR analysis.
